# Urachal carcinoma: Impact of recurrence pattern and lymphadenectomy on long‐term outcomes

**DOI:** 10.1002/cam4.3059

**Published:** 2020-04-23

**Authors:** Fangfang Duan, Wenyu Zhai, Bei Zhang, Shengjie Guo

**Affiliations:** ^1^ VIP Region State Key Laboratory of Oncology in South China Collaborative Innovation Center for Cancer Medicine Sun Yat‐sen University Cancer Center Guangzhou P.R. China; ^2^ Department of Thoracic Surgery State Key Laboratory of Oncology in South China Collaborative Innovation Center for Cancer Medicine Sun Yat‐sen University Cancer Center Guangzhou P.R. China; ^3^ Department of Urology Surgery State Key Laboratory of Oncology in South China Collaborative Innovation Center for Cancer Medicine Sun Yat‐sen University Cancer Center Guangzhou P.R. China

**Keywords:** lymphadenectomy, prognostic factors, recurrence pattern, Urachal carcinoma

## Abstract

**Background:**

Urachal carcinoma is a rare nonurothelial malignant tumor with high rates of local recurrence and systemic metastasis. Although radical resection is widely considered the standard treatment, there is still a debate regarding the benefits of lymphadenectomy. To explore these factors, we investigated the recurrence pattern of urachal cancer and the impact of lymphadenectomy on long‐term survival.

**Methods:**

The data of 62 patients pathologically diagnosed with urachal carcinoma at Sun Yat‐sen University Cancer Center from 2002 to 2019 were retrospectively reviewed. Lymphadenectomy was defined as lymph nodes retrieved from the obturator, internal iliac, and external iliac lymph node stations. The Kaplan‐Meier method and Cox regression model were used to identify prognostic factors. OS and DFS were the primary endpoints.

**Results:**

Of the 47 males and 15 females included, 54 patients underwent partial cystectomy, and 27 patients underwent lymphadenectomy. The number of patients with Sheldon stage IIIA, IIIB, IIIC, IVA, and IVB were 43 (69.4%), 4 (6.5%) 3 (4.8%), 6 (9.7%), and 6 (9.7%), respectively. The median DFS was 32.7 months, and the mean OS was 114.6 months. Sheldon stage (*P* < .001) and tumor size (*P* = .001) were identified as independent prognostic factors for DFS, whereas Sheldon stage (*P* = .003), peritoneal metastasis (*P* = .006), distant metastasis (*P* = .024), and recurrence in pelvic lymph nodes (*P* = .015) were independent prognostic factors for OS.

**Conclusions:**

Urachal carcinoma has a high recurrence rate, but only peritoneal metastasis, distant metastasis, and recurrence in pelvic lymph nodes were found to be associated with OS. Lymphadenectomy was recommended because of its role in accurately staging the disease, and further research is needed to focus on lymphadenectomy and standardized the procedure.

## Graphical abstract

Urachal carcinoma has a high recurrence rate, but only peritoneal metastasis, distant metastasis, and recurrence in pelvic lymph nodes were found to be associated with OS. Although lymphadenectomy was not an independent prognostic factor for DFS and OS, the data of this study showed a possibility that lymphadenectomy could improve surgical outcomes.

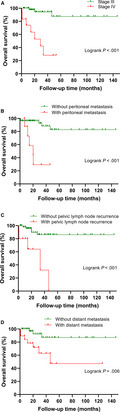

## 1 INTRODUCTION

Urachal carcinoma, an uncommon nonurothelial tumor that most often occurs at the junction of the urachal ligament and urinary bladder dome or anywhere along with the midline of the bladder, constitutes less than 1% of all urinary bladder tumors and primarily occurs in males.^1^, ^2^, ^3^, ^4^, ^5^ Several published reports have shown that adenocarcinomas, including mucinous adenocarcinoma, intestinal signet‐ring cell carcinoma, and mixed signet‐ring cell carcinoma, account for nearly 90% of urachal carcinomas. However, only a few squamous cell carcinomas, transitional epithelial carcinomas, sarcomas, and undifferentiated carcinomas have been reported.^3^, ^6^, ^7^ Due to the lack of early clinical symptoms,^9^ patients with urachal carcinoma are usually diagnosed when the disease is already at an advanced stage and is often accompanied by local extension (resulting in local recurrences) and systemic metastasis (resulting in death) before any related medical intervention is even started.^8^, ^9^, ^10^

Currently, surgery, including partial resection or radical resection, remains the mainstay therapy because it can lead to a median survival of 48 months and 5‐year survival rates of 45%‐49%.^3^, ^11^, ^12^ Despite recent published literature reporting no significant survival differences between partial and complete resection,^1^, ^11^, ^13^ compared to radical cystectomy, partial cystectomy has been shown to have superior oncological safety to complete resection while maintaining similar efficacy. Additionally, partial cystectomy is also accompanied by a better quality of life because of fewer postoperative complications.^13^ Therefore, partial cystectomy with en bloc excision of the urachal ligament, umbilicus, and dome of the bladder is most commonly recommended in clinical practice.^2^, ^3^, ^10^

However, whether lymphadenectomy is of any benefit to patients with urachal carcinoma is still highly debatable.^1^, ^5^ Some surgeons support lymphadenectomy, as it can lead to the perioperative excision of occult lymph node metastasis, which has been related to improved survival, thereby achieving an increase in the 5‐year survival rate by 25%.^11^ Others insist that performing aggressive surgical resection due to the presence of enlarged lymph nodes has no significant survival benefit.^3^, ^13^ Given the rarity of this tumor and the absence of large‐scale studies, reaching a consensus on such topics remains challenging.

To explore this dilemma, we retrospectively reviewed all patients diagnosed and treated with urachal carcinoma at Sun Yat‐sen University Cancer Center during the last 20 years and investigated any patterns of recurrence. Moreover, we explored the impact of lymphadenectomy on the long‐term survival of patients with urachal carcinoma.

## 2 MATERIALS AND METHODS

### 2.1 Patient cohort

After obtaining approval from the ethics committee of Sun Yat‐sen University Cancer Center, we identified the records of 82 patients who presented urachal masses from 2002 to 2019. A total of 62 were pathologically diagnosed as urachal carcinoma, while of the remaining cases, nine were grossly diagnosed as inflammation, three were benign urachal lesions, six were pathologically diagnosed as bladder carcinoma, one was diagnosed as sigmoid cancer, and one was discharged early from hospital due to active pulmonary tuberculosis and his subsequent treatment data could not be retrieved.

### 2.2 Surgical approach

Radical or partial cystectomy with excision of the urachal ligament and umbilicus was defined as radical resection. Cytoreductive surgery and peritoneal nodule biopsy were defined as nonradical resection. Lymphadenectomy was defined as lymph nodes retrieved from the obturator, internal iliac, and external iliac lymph node stations.

### 2.3 Pathologic review and staging

A central pathologic assessment was carried out by at least two pathologists. Urachal carcinoma was classified as poor, moderate, or well differentiated. The primary histologic cell type, as well as any significant secondary histologic features, was also recorded. In addition, tumors at diagnosis were retrospectively staged according to the Sheldon staging system,^1^ classifying the tumors as stage I, tumor confined to the urachal mucosa; stage II, tumor with invasion confined to the urachus itself; stage IIIA, tumor with local extension to the bladder; stage IIIB, tumor with local extension to the abdominal wall; stage IIIC, tumor invading the peritoneum; stage IIID, tumor invading local viscera other than the bladder; stage IVA, urachal cancer with metastasis to the lymph nodes; and stage IVB, urachal cancer with distant metastases.

### 2.4 Follow‐up and survival analysis

The regular follow‐up after the operation included visits to an outpatient clinic at 3‐month intervals for the first 2 years and every 6 months in subsequent years. For patients who did not visit the clinic, follow‐up information was obtained by telephone call. Patients underwent routine blood and biochemical analyses, physical examination, and abdominal and pelvic computed tomography (CT) scans. When there was any evidence of suspected recurrence, chest, abdominal, and pelvic CT scans; brain magnetic resonance imaging (MRI); bone scintigraphy; and positron emission tomography were performed. A diagnosis of recurrence was in accordance with relevant diagnostic imaging or cytological/histologic findings. The calculation of the patients’ disease‐free survival (DFS) and overall survival (OS) was carried out as our primary endpoint. DFS was defined from the time of definitive diagnosis to the day of first recurrence confirmed by radiological scan or biopsy, with detailed mention of the recurrent site. OS was calculated from the date of diagnosis to the date of death or last follow‐up visit.

### 2.5 Statistical analysis

Fisher's exact test was used to compare categorical data, such as lymph node dissection, pathological pelvic lymph node metastasis, postoperative pelvic lymph node stage, and postoperative pelvic lymph node recurrence. Univariate and multivariate Cox proportional hazards regression models were established to identify prognostic and independent factors. Covariates with a *P* < .1 in the univariate analysis were used for the multivariate analysis. DFS and OS were calculated using the Kaplan‐Meier analysis method, and comparisons of survival between different groups were assessed by the log‐rank test. Statistical analyses were performed using SPSS software, version 22 (SPSS Inc), and *P* < .05 was considered statistically significant.

## 3 RESULTS

### 3.1 Patient characteristics

The characteristics of the 62 enrolled patients are listed in Table 1. There were 47 males and 15 females included with a median age of 51.5 ± 13.2 (range 22 to 69) years. Forty‐five (72.6%) patients had naked‐eye hematuria, eight (12.9%) had urinary irritation, 14 (22.6%) presented with an abdominal mass, two (3.2%) had omphalorrhoea, and two (3.2%) were diagnosed upon general physical examination without any initial presenting symptoms. All patients underwent a preoperative CT, MRI, or urological ultrasound scan. The clinical median tumor diameter was 3.75 ± 2.28 cm. All of the patients underwent surgery, including 54 of 62 (87%) treated with partial resection and 43.5% (27 of 62) with lymph node dissection. The number of patients with lymph nodes dissected at stations 1, 2, 3, and 4 was 3, 8, 4, and 12, respectively. The postoperative histopathological diagnosis indicated that 95.4% (59 of 62) and 40.3% (25 of 62) of cases were well‐ or moderately differentiated carcinoma, respectively. According to the Sheldon staging system for urachal carcinoma,^1^ 69.4% (43 of 62) had local extension to the bladder (stage IIIA), 6.5% (4 of 62) exhibited extension to the abdominal wall (stage IIIB), 4.8% (3 of 62) invaded the peritoneum (stage IIIC), and 33.9% (21 of 62) had stage IV disease, including 6 (9.7%) with regional lymph node metastasis and 6 (9.7%) presented with distant metastases. Elevations in carcinoembryonic antigen (CEA), CA19‐9, CA125, CA724, CyFra21‐1, and neuron‐specific enolase (NSE) were observed in some patients (21%, 8.1%, 1.6%, 19.4%, 14.5%, and 4.8%, respectively).

**TABLE 1:** Characteristics of 62 patients with Urachal carcinoma

Characteristics	Median or no. of cases (%)
Gender	
Male	47 (75.8)
Female	15 (24.2)
Age (y)	51.5 ± 13.2
Tumor size (cm)	3.75 ± 2.28
Hematuria	
No	17 (27.4)
Yes	45 (72.6)
Urinary irritation	
No	54 (87.1)
Yes	8 (12.9)
Omphalorrhoea	
No	60 (96.8)
Yes	2 (3.2)
Abdominal mass	
No	48 (77.4)
Yes	14 (22.6)
Tumor calcification in imaging	
No	55 (88.7)
Yes	7 (11.3)
Preoperative metastasis	
No	51 (82.3)
Pelvic lymph node	4 (6.5)
Peritoneal metastasis	2 (3.2)
Abdominal wall metastasis	2 (3.2)
Lung and mediastinal lymph node metastasis	3 (4.8)
Sheldon stage	
IIIA	43 (69.4)
IIIB	4 (6.5)
IIIC	3 (4.8)
IVA	6 (9.7)
IVB	6 (9.7)
Histology	
Adenocarcinoma	59 (95.4)
Others	3 (4.6)
Differentiation degree	
Well or moderate	25 (40.3)
Poor	17 (27.4)
Not mentioned	20 (32.3)
Vascular invasion	
Positive	7 (11.3)
Negative	22 (35.5)
Not mentioned	33 (53.2)
Operative approach	
Partial resection	54 (87.0)
Radical resection	8 (13.0)
Lymph node dissected	
Yes	27 (43.5)
No	35 (56.5)
Postoperative chemotherapy	
No	44 (71.0)
Yes	18 (29.0)
Positive lymph node stage	
Yes	6 (9.7)
No	56 (90.3)
The second operation after recurrence	
No	50 (80.6)
Yes	12 (19.4)
CEA	
Abnormal	13 (21)
Normal	26 (41.9)
Not mentioned	26 (37.1)
CA199	
Abnormal	5 (8.1)
Normal	32 (51.6)
Not mentioned	27 (40.3)
CA125	
Abnormal	1 (1.6)
Normal	14 (22.)
Not mentioned	46 (75.8)
CA724	
Abnormal	12 (19.4)
Normal	20 (32.3)
Not mentioned	30 (48.4)
Abnormal NSE	3 (4.8)
Abnormal CyFra21‐1	9 (14.5)
At least 2 abnormal serum tumor markers	14 (22.6)
CK20	
Positive	38 (61.3)
Negative	2 (3.2)
Not mentioned	22 (35.5)
CK7	
Positive	20 (32.3)
Negative	15 (24.2)
Not mentioned	27 (43.5)
Nuclearβ	
Positive	17 (27.4)
Negative	45 (72.6)
CDX‐2	
Positive	34 (54.8)
Negative	1 (1.6)
Not mentioned	27 (43.5)

### 3.2 Survival and prognostic factors of survival

**Figure 1:**
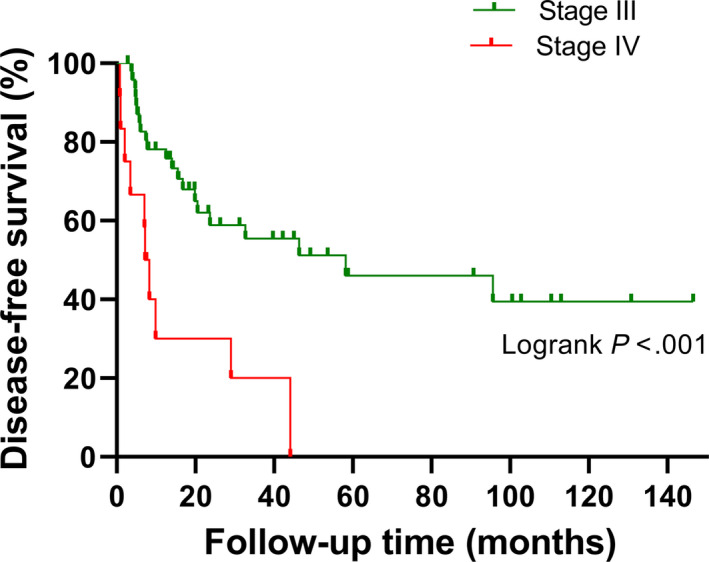
Kaplan‐Meier analysis of disease‐free survival for patients with urachal carcinoma in the Sheldon stage III and those in the Sheldon stage IV (*P* ＜ .001)

**Figure 2:**
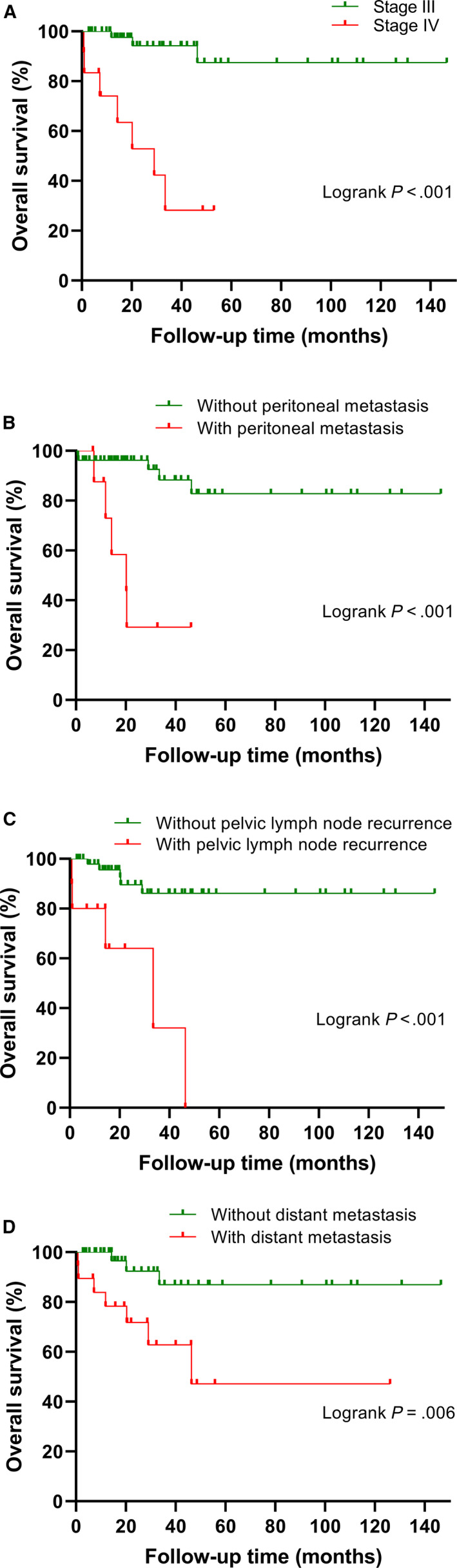
Kaplan‐Meier analysis of overall survival for patients with urachal carcinoma. A, Patients with stage IV versus stage III. B, Patients with peritoneal metastasis versus without peritoneal metastasis. C, Patients with pelvic lymph node recurrence versus without pelvic lymph node recurrence. D, Patients with distant metastasis versus without distant metastasis

The median DFS for the entire study cohort was 32.7 months. Sheldon stage and tumor size were independent prognostic factors for DFS (*P* < .001, HR = 5.896, 95% CI 2.286‐15.208; *P* = .002, HR = 1.388, 95% CI 1.126‐1.710, respectively) (Table 2). Interestingly, although the operative approach was associated with DFS (*P* = .002), it was not statistically significant in the multivariate analysis. Significant survival differences in DFS and OS were found between Sheldon stages III and IV (*P* < .001, Figure 1; *P* < .001, Figure 2A).

**TABLE 2:** Prognostic factors of the disease‐free survival and overall survival for patients with Urachal carcinoma

Factors	Univariate analysis		Multivariate analysis	
	HR (95%CI)	*P* value	HR (95%CI)	*P* value
*Prognostic factors of DFS*				
Gender	0.712 (0.292‐1.736)	.455		
Age (y)	1.008 (0.980‐1.038)	.575		
Tumor size (cm)	1.340 (1.099‐1.633)	.004	1.388 (1.126‐1.710)	.002
Sheldon stage (III vs IV)	3.609 (1.651‐7.889)	.001	5.896 (2.286‐15.208)	<.001
Preoperative metastasis	3.168 (1.430‐7.019)	.005		
Differentiation degree				
Well or moderate	Ref			
Poor	0.830 (0.359‐1.922)	.664		
Not mentioned	0.857 (0.355‐2.068)	.732		
Vascular invasion				
No	Ref			
Yes	1.543 (0.538‐4.426)	.420		
Not mentioned	0.381 (0.168‐0.865)	.021		
Lymph node dissected	1.269 (0.599‐2.690)	.534		
Operative approach	1.638 (1,202‐2.333)	.002		
Positive lymph node stage	1.815 (0.688‐4.787)	.229		
*Prognostic factors of OS*				
Gender	1.445 (0.372‐5.615)	.595		
Age (y)	0.986 (0.939‐1.036)	.577		
Tumor size (cm)	1.376 (1.077‐1.7559)	.011		
Sheldon stage (III vs IV)	12.408 (3.196‐48.164)	<.001	12.523 (2.433‐64.460)	.002
Preoperative metastasis	9.856 (2.761‐35.188)	<.001		
Differentiation degree				
Well or moderate	Ref			
Poor	0.465 (0.094‐2.310)	.349		
Not mentioned	0.564 (0.122‐3.145)	.564		
Vascular invasion				
No	Ref			
Yes	1.732 (0.310‐9.675)	.532		
Not mentioned	0.446 (0.110‐1.802)	.257		
Lymph node dissected	2.147 (0.599‐7.602)	.241		
Operative approach	1.963 (1.281‐3.010)	.002		
Positive lymph node stage	5.606 (1.576‐20.056)	.008		
Bladder recurrence	2.631 (0.722‐9.444)	.138		
Abdominal wall recurrence	3.098 (0.642‐14.947)	.159		
Postoperative peritoneal metastasis	10.693 (2.789‐40.991)	.001	9.999 (1.906‐52.468)	.006
Postoperative pelvic lymph node recurrence	8.677 (2.469‐30.493)	.001	7.024 (1.429‐34.519)	.016
Postoperative distant metastasis	1.397 (21.022)	.015	8.416 (1.287‐55.036)	.026
Postoperative chemotherapy	1.407 (0.394‐5.027)	.599		
The second operation after recurrence	0.976 (0.206‐4.620)	.976		

Table 2 shows that Sheldon stage (*P* = .002, HR = 12.523, 95% CI 2.433‐64.460), postoperative peritoneal metastasis (*P* = .006, HR = 9.999, 95% CI 1.960‐53.539), postoperative distant metastasis (*P* = .028, HR = 8.416, 95% CI 1.287‐55.036), and recurrence in pelvic lymph nodes (*P* = .016, HR = 7.024, 95% CI 1.429‐34.519) were independent prognostic factors for impaired OS. Furthermore, we found that patients without peritoneal or distant metastasis had a significantly better OS than those with peritoneal or distant metastasis (*P* < .001, Figure 2B; *P* = .006, Figure 2D).

### 3.3 Relapse

Up to August 30, 2019, 31 of the initial 62 patients (50%) were alive and free of disease after surgical resection (median follow‐up 38.8 months). Conversely, 31 patients (50%) had metastases. The median time to recurrence after the primary surgery was 32.7 months. The sites of metastases are detailed in Table 3. Fifteen (24.2%) patients had metastases in their bladder, and the number of patients with metastasis in the abdominal wall, intraperitoneal cavity, and pelvic lymph nodes was 5 (8.1%), 10 (16.1%), and 10 (16.1%), respectively. Lung and mediastinal lymph nodes accounted for the majority of postoperative metastases (30.6%; 19 of 62 patients).

**TABLE 3:** Sites of metastases in 31 patients after the primary resection

The location of recurrence or metastasis	Median or case no. (%)
Bladder	15 (24.2)
Abdominal wall	5 (8.1)
Peritoneal metastasis	10 (16.1)
Pelvic lymph node	10 (16.1)
Distant metastasis	19 (30.6)
Lung and mediastinal lymph nodes	19 (30.6)
Bone	1 (1.6)
Liver	2 (3.2)
Total	31 (50)

**Figure 3:**
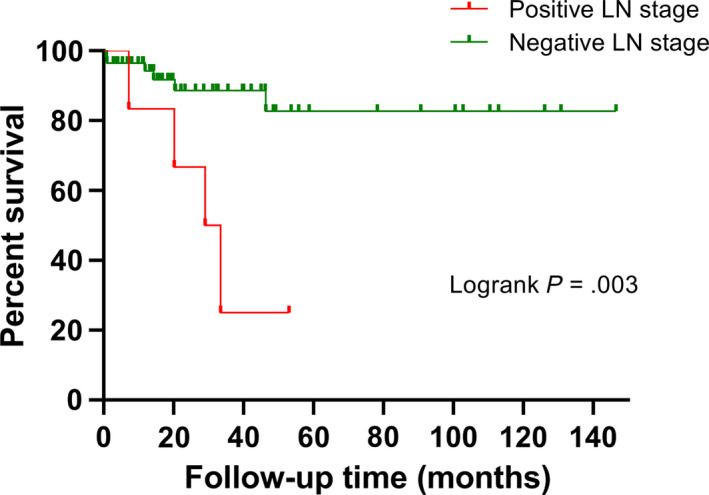
Kaplan‐Meier analysis of survival for patients with positive lymph node and patents just with negative lymph node stage (*P* = .003)

The data from Table 4 indicate that 6.9% (4 of 58) of the patients had pathologically confirmed metastases in their lymph nodes, although they were diagnosed without pelvic lymph node metastasis before their surgery. Half of these patients (2 of 4 patients), who were thought to have metastasis in the pelvic lymph nodes before the surgery, were confirmed as negative for lymph node metastasis. A statistically significant difference was observed between them (Fisher's exact test *P* = .043). Subsequently, we found that the survival between patients with positive lymph node staging and those with negative lymph node staging was significantly different (*P* = .003, Figure 3). As shown in Table 5, compared with patients who underwent pelvic lymph node dissection, numerically, patients without pelvic lymph node dissection experienced a higher rate of postoperative pelvic lymph node recurrence (11.1% vs 20%), even though statistical significance was not achieved (Fisher's exact test *P* = .491). However, compared to patients with pelvic lymph node recurrence, patients without pelvic lymph node recurrence had significantly superior OS (*P* < .001, Figure 2C).

**TABLE 4:** The correlation between with‐without pelvic lymph node metastasis and postoperatively pelvic lymph node stage positive or negative

Group	Positive lymph node stage	Negative lymph node stage	Total
With pelvic lymph node metastasis	2	2	4
Without pelvic lymph node metastasis	4	54	58
Total	6	56	62

Note

Fisher's Exact Test *P* = .043.

**TABLE 5:** The correlation between with‐without pelvic lymph node dissected and with‐without postoperatively pelvic lymph node recurrence

Group	Without pelvic lymph node recurrence	With pelvic lymph node recurrence	Total
Without pelvic lymph node dissected	28	7	35
With pelvic lymph node dissected	24	3	27
Total	52	10	62

Note

Fisher's Exact Test *P* = .491.

## 4 DISCUSSION

In this study, we retrospectively reviewed patients with confirmed urachal carcinoma who were treated at Sun Yat‐sen University Cancer Center during the last 20 years, comprehensively presented their clinical and pathological features and presented several original findings.

Elevations in some tumor markers, such as CEA, CA19‐9, and CA125, were observed in some patients in this study (12 of 32 patients, five of 37, and one of 15, respectively). Arlene et al reviewed 42 patients from the MD Anderson Cancer Center and found that 13 of 22 patients had increased CEA levels, 6 of 10 patients had increased CA19‐9 levels, and 7 of 16 patients had increased CA125 levels.^5^ Although the cohort in MD Anderson Cancer Center had a higher percentage of patients with elevated tumor markers, both studies revealed the clinical similarities of urachal cancer and gastroenteric cancer. Most importantly, elevated CA724 levels were found in 37.5% (12/32) of preoperative patients, which revealed the potential value of CA724 in diagnosis. One case report about urachal carcinoma reported that increased CA724 was observed in a patient with local recurrence and ovary metastasis after resection, which showed value of detection of postoperative recurrence.^14^ CA724 is a useful marker for predicting the efficacy of treatment in gastric cancer, and its prognostic value has been widely recognized.^15^, ^16^ Thus, given the established value of CA724 in diagnosing, staging, and prognosticating gastric cancer, we surmise the potential value of CA724 in guiding the clinical practice of urachal carcinoma, which needs further study. Additionally, similar to the immunophenotypic features in gastroenteric tumors reported by Paner et al,^17^ there may be a certain similarity between urachal carcinoma and gastrointestinal neoplasms in terms of cellular structures and biological behaviors, to a certain extent. At the same time, patients with urachal carcinoma may also benefit from chemotherapy for gastrointestinal tumors. Two previous studies have revealed that chemotherapy regimens combining 5‐FU and cisplatin showed better therapeutic effects.^5^, ^18^ Unfortunately, the study failed to show the survival advantage of chemotherapy because of a lack of relevant data. We look forward to more advances in chemotherapy for urachal carcinoma.

Our study findings also demonstrated the preferred pattern in the site of recurrence after primary resection. In their meta‐analysis, Tibor Szarvas et al mentioned that a considerable proportion of patients develop distant metastases and lymph node metastases, with the lung, bone, and peritoneum as the most common sites of metastasis.^18^ In the cohort from the MD Anderson Cancer Center, bone (13 of 26 patients), lung (12 of 26 patients), and liver (7 of 26 patients) were the three most common sites of metastasis.^5^ In this study, lung and mediastinal lymph node metastasis, recurrence in the bladder, pelvic lymph node metastasis, and peritoneal metastasis were observed in 19, 15, 10, and 10 of 31 patients, respectively. These data may help oncologists monitor the progression and relapse of urachal cancer. Moreover, the multivariate analysis of OS found that some recurrence patterns had a more negative effect on survival. Peritoneal metastasis, distant metastasis, and recurrence in the pelvic lymph nodes were independent prognostic factors for OS. Compared with other patterns of recurrence, recurrence in the bladder and abdominal wall had a limited influence on OS. Although undergoing a second operation showed no statistical significance in the Cox regression model, one possible explanation is that recurrence in the bladder and abdominal wall is considered local recurrence, and patients still have a chance to completely remove these lesions during the second operation after their relapse. Distant metastasis is the most common recurrence pattern and is strongly associated with worse long‐term survival, which clearly underlines the need for effective systemic therapy.

Another key finding of this study is the role of lymphadenectomy. There is still no agreement about whether lymphadenectomy is beneficial to patients with urachal cancer. On the one hand, there is no study proving that lymphadenectomy can improve long‐term outcomes. One report reviewed 152 patients, which included 43 patients who underwent lymphadenectomy, and did not observe a positive effect of lymphadenectomy on survival.^19^ Niedworok et al reviewed 26 patients and revealed that lymphadenectomy was not a prognostic factor of OS or progression‐free survival.^20^ The rare positive rate of lymph node metastases and the limited number of patients in a single‐center study might explain why lymphadenectomy was not a prognostic factor. In addition, a lack of consensus on the lymphadenectomy procedure may cause discrepancies in its quality, which might have weakened its effect on OS. On the other hand, lymphadenectomy plays an important role in accurately staging urachal carcinoma. Given the general fact that preoperative imaging cannot detect all invaded lymph nodes in many kinds of cancers,^21^, ^22^, ^23^, ^24^, ^25^, ^26^ except for the novel findings mentioned above, we also emphasized exploring whether patients presenting obturator, internal iliac, and external iliac lymph node metastasis on imaging truly had metastasis by comparing the images to the pathology after resection. In this study, two of four patients who were clinically diagnosed with node‐positive stage disease were pathologically diagnosed with node‐negative stage disease, and four patients who were clinically diagnosed with node‐negative stage disease were pathologically diagnosed with node‐positive stage disease. Although lymphadenectomy was not an independent prognostic factor for DFS or OS, it could improve surgical outcomes. High‐quality lymphadenectomy can remove lymph nodes with micrometastases, and patients benefit from removing positive lymph nodes.

In bladder urothelial carcinoma and gastrointestinal tumors, lymph node dissection has been shown to be significant for accurately staging the disease and improving patient prognosis.^27^, ^28^, ^29^ Given that urachal carcinoma has a similar location to bladder urothelial carcinoma and a similar histology to gastrointestinal tumors in histology, we strongly believe that pelvic lymph node dissection during the primary resection of urachal carcinoma is beneficial for patients, although we failed to find a significant predictive value of pelvic lymph node dissection in urachal carcinoma in this study.

Despite the innovation of this study as mentioned above, there are still some limitations that should be considered. The principal limitation is its small sample size and retrospective nature, and bias inevitably existed. Furthermore, loss of immunohistochemistry data in some cases limits the statistical power of this study. In addition, the lack of relevant chemotherapy data may affect the reliability of the results. However, this is a dilemma that all health‐care providers who treat rare diseases must confront. We look forward to pooling data from multiple centers into a larger common dataset in the future to carry out larger studies of urachal carcinoma.

## 5 CONCLUSIONS

In summary, our findings showed that the Sheldon stage, peritoneal metastasis, distant metastasis, and recurrence in pelvic lymph nodes were associated with long‐term survival. Although lymphadenectomy was not an independent prognostic factor for DFS and OS, lymphadenectomy was recommended because of its role in accurate staging. Further research is needed to focus on lymphadenectomy and make it standardized.

## CONFLICT OF INTEREST

None.

## AUTHOR CONTRIBUTIONS

Fangfang Duan gathered data. Wenyu Zhai performed data analyses. Wenyu Zhai processed the figures. Fangfang Duan wrote the manuscript. Fangfang Duan, Wenyu Zhai, and Bei Zhang revised the manuscript. Shengjie Guo and Bei Zhang designed the study. All authors approved the final version of the manuscript.

## Data Availability

The key raw data have been deposited into the Research Data Deposit (http://www.researchdata.org.cn), with the Approval Number of RDDA2020001354, and the datasets used in this study are publicly available.
